# Lithium deficiency and Alzheimer’s: emerging evidence and therapeutic implications

**DOI:** 10.1097/MS9.0000000000004123

**Published:** 2025-10-17

**Authors:** Amisha Kumari, Reena Kumari, Muddassir Khalid, Supoma Ghosh Ria

**Affiliations:** a Department of Medicine, Liaquat University of Medical and Health Sciences, Jamshoro, Pakistan; b Department of Medicine, Peoples University of Medical and Health Sciences for Women, Nawabshah, Pakistan; c Department of Medicine, Nishtar Medical University, Multan, Pakistan; d Mugda Medical College, University of Dhaka, Dhaka, Bangladesh

*Dear Editor,*

Alzheimer’s disease (AD) remains a growing international health menace, and there is a dearth of disease-modifying treatments. There has been emergent evidence to suggest that at trace levels, which apply to the brain and not to be confused with psychiatric dosage levels, lithium could have an underestimated neuroprotective effect. A seminal study in the journal *Nature* (2025) has shown that lithium is targeted in the early stages of AD, is transported by amyloid 2, and that the restoration of brain-available lithium levels can reverse cognitive decline in mouse models^[1]^.

These mechanistic findings are corroborated by a 2024 meta-analysis of clinical epidemiological evidence indicating that lithium treatment is linked to a significant decrease in the risk of AD [relative risk (RR) 0.59; 95% confidence interval (CI) 0.44–0.78] and of dementia more generally (RR 0.66; 95% CI 0.56–0.77)^[2]^. Also, a systematic review and meta-analysis conducted in 2024 suggested that lithium was superior to high-cost antiamyloid therapies, including aducanumab and lecanemab, in network analyses of cognitive outcomes in people with mild cognitive impairment and AD^[3]^.

Positive clinical trials have also been achieved. Subtherapeutic doses of lithium carbonate (target 0.25–0.5mEq/L) were demonstrated to maintain cognitive functioning and suppress cerebrospinal fluid biomarkers in patients with amnestic mild cognitive impairment, and to provide greater stability than placebo in a 2-year, double-blind, randomized, placebo-controlled trial^[4]^. Also, a randomized controlled trial in patients with AD (Lit-AD) assessed the behavioral comorbidities of low-dose lithium, which showed tolerability and suggestive treatment value^[5]^.

Collectively, recent mechanistic, epidemiological, and clinical evidence, including randomized trials, meta-analyses, and high-impact preclinical discoveries, cement the candidacy of lithium as a disease-modifying agent in AD. Since lithium is inexpensive and has a stable biological plausibility, the field should rapidly proceed to large-scale, biomarker-directed, randomized controlled studies using micro- to subtherapeutic dosing. Such studies are to focus on cognitive outcomes, renal and thyroid safety monitoring, and stratification of the risks of developing AD (e.g., APOE ε4 status and biomarker profile). In the meantime, mapping local exposure to lithium in water and diet could be used to inform prevention efforts.

Should it prove viable, low-dose lithium might act as a safe, scalable preventive or early-intervention weapon, similar to that of iodine in salt, and introduce palpable promise to the war on dementia.

TITAN Guidelines: This manuscript complies with TITAN Guidelines, 2025, declaring no use of artificial intelligence^[6]^.

## Data Availability

Not applicable.

## Peer and provenance statement

Not commissioned, externally peer-reviewed.
